# Membrane Bound GSK-3 Activates Wnt Signaling through Disheveled and Arrow

**DOI:** 10.1371/journal.pone.0121879

**Published:** 2015-04-07

**Authors:** Anirudh G. Mannava, Nicholas S. Tolwinski

**Affiliations:** Yale-NUS College and Department of Biological Sciences, National University of Singapore, Block MD6, Centre for Translational Medicine, Yong Loo Lin School of Medicine, 14 Medical Drive, Level 10 South, 10-02M, Singapore 117599, Singapore; Simon Fraser University, CANADA

## Abstract

Wnt ligands and their downstream pathway components coordinate many developmental and cellular processes. In adults, they regulate tissue homeostasis through regulation of stem cells. Mechanistically, signal transduction through this pathway is complicated by pathway components having both positive and negative roles in signal propagation. Here we examine the positive role of GSK-3/Zw3 in promoting signal transduction at the plasma membrane. We find that targeting GSK-3 to the plasma membrane activates signaling in Drosophila embryos. This activation requires the presence of the co-receptor Arrow-LRP5/6 and the pathway activating protein Disheveled. Our results provide genetic evidence for evolutionarily conserved, separable roles for GSK-3 at the membrane and in the cytosol, and are consistent with a model where the complex cycles from cytosol to membrane in order to promote signaling at the membrane and to prevent it in the cytosol.

## Introduction

The Wnt or Wingless (Wg in Drosophila) signaling pathway is essential for the proper development of animals. Wnt signals control cell differentiation, proliferation, migration, polarity, and patterning [1,2]. In humans, Wnt components have been found to affect stem cell maintenance and tumor progression [1,2]. There are several types of Wnt pathways, including polarity determination and ion concentration branches [3,4]. Here we concentrate on the canonical branch of signaling, where the basic step is the regulation of Armadillo/β-catenin (Arm/β-cat) protein levels. When the pathway is active, Arm protein levels increase followed by translocation to the nucleus and transcriptional activation. In the absence of ligand, the pathway is turned off by the formation of a degradation complex consisting of the scaffold proteins Axin and APC and the kinases CKI and GSK-3 (Shaggy, Zw3). This complex controls the phosphorylation state of Arm with N-terminal phosphorylation tagging it for proteasome-mediated degradation. When signaling is activated, Wnt binding initiates the movement of the destruction complex to the plasma membrane where it becomes the activating complex adding the transmembrane receptors Frizzled (Fz) and Arrow (LRP5/6, Arr) and the signaling protein Disheveled (Dsh). This complex activates signaling by counteracting the destruction of Arm causing increased Arm protein levels. Arm in turn translocates to the nucleus where it activates transcription in conjunction with the transcription factor TCF [2,5,6].

The Wnt ligands were discovered over 30 years ago [7], but the pathway mechanism was established through genetic screens in the late 1980’s [8–10], biochemistry, genetic epistasis, and cancer cell studies starting in the early 1990’s [11–14]. The membrane-proximal activating complex, however, is more recent. The key discovery underpinning this complex was an unexpected activating function of GSK-3 when expressed in a membrane-tethered form [15]. Previously, the membrane-proximal events of Wnt signal transduction were poorly understood. The discovery of a positive role for GSK-3 went some way to bridge the gap between ligand binding and destruction complex inhibition. The current model posits a mechanism where ligand mediated receptor activation leads to GSK-3 mediated phosphorylation of Arrow on PPPSPxS motifs creating binding sites for Axin disrupting the destruction complex [15,16]. This is an important advance as Axin appears to be the rate-limiting component, and its levels are regulated through proteasomal degradation in a signal dependent manner [17–19].

Here we report that a membrane-tethered form of GSK-3 activates Wnt signaling in Drosophila embryos. We use epistasis to characterize the pathway position of membrane-tethered GSK-3 as compared to untethered GSK-3. We find that membrane-tethered GSK-3 is unable to activate signaling unless functional copies of Arrow and Dsh are present. These results support a model where a membrane-proximal complex must form in order for signal to be transmitted.

## Results

### Membrane tethered GSK-3 activates signaling

**Fig 1:**
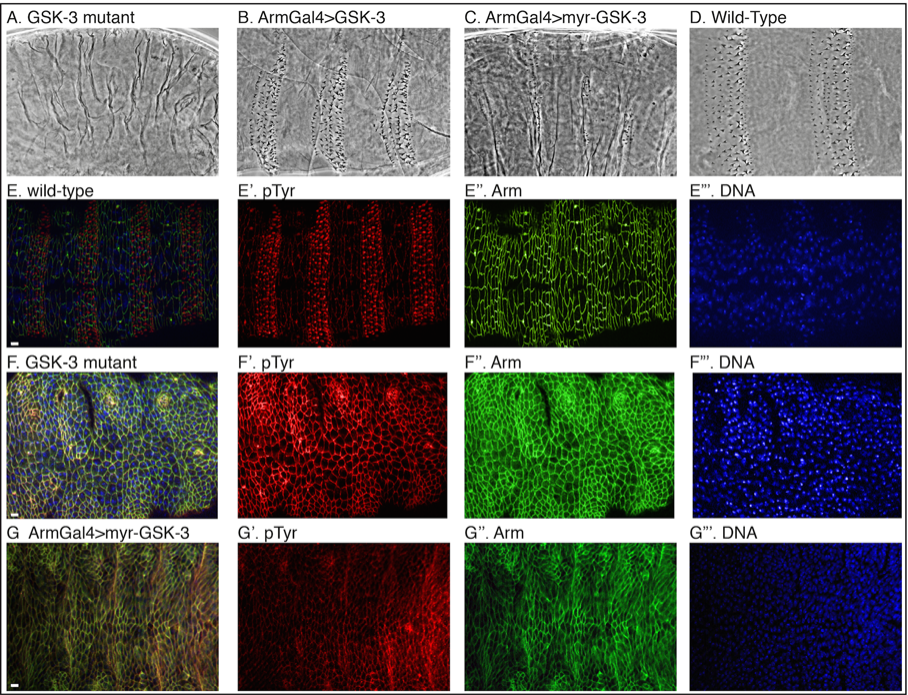
Expression of membrane-tethered GSK-3 activates Wnt signaling. (A) Loss of GSK-3 (*zw3* ^*M11-1*^ germline clones maternally and zygotically mutant) embryos show hyper-activated Wnt signaling or the naked phenotype. (B) Overexpression of GSK-3 has no effect on cuticle patterning. (C) Expression of membrane-tethered myr-GSK-3 shows the hyper-activated Wnt signaling or the naked phenotype. (D) Wild-type cuticle for comparison. (E-E”‘) A wild-type embryo at stage ~15 with junctions and cell outlines in green (Arm) and denticle precursors in red (pTyr). (F-F”‘) Similar stage embryo (M/Z) mutant for GSK-3 shows no denticle precursors. (G-G”‘) Membrane-tethered GSK-3 expression also prevents denticle precursors from forming. Scale bar = 10μm.

**Fig 2:**
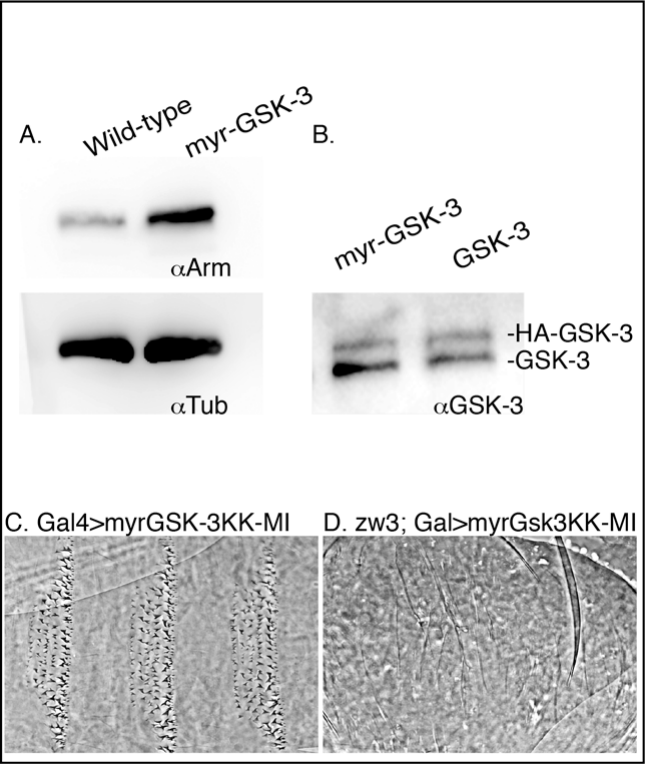
Expression of membrane-tethered GSK-3 increases embryonic levels of Arm. (A) Western blot comparing total Arm protein levels between five embryos expressing myr-GSK-3 and 5 wild-type embryos. (B) Western blot comparing total GSK-3 protein levels. Higher band represents expressed GSK-3 as this has a 3XHA tag, making it slightly larger than the endogenous GSK-3 directly below. (C) Cuticle of embryo expressing myr-GSK-3 kinase dead variant using ArmGal4. (D) Cuticle of embryo expressing myr-GSK-3 kinase dead variant using ArmGal4 in an embryo maternally and zygotically mutant for GSK-3.

GSK-3 and CKI comprise a dual kinase phosphorylation mechanism activating Arm degradation and turning off signaling [20,21]. Upstream, GSK-3 and CKI phosphorylate Arrow and turn on signaling [15]. The former function is epistatic to the latter, and GSK-3 mutants have a strong “naked” phenotype coinciding with Wnt pathway hyper-activation (Fig 1A) [13,22,23]. Loss of GSK-3 in Drosophila embryos results in high levels of Arm protein. This loss of function phenotype and pathway activation can be rescued with the overexpression of a wild-type form of GSK-3, but not a kinase deficient form (Table 1, and [24]). In wild-type adult fly tissues, over expression of GSK-3 can block signaling whereas a kinase dead form has no effect [24], but this does not occur in embryos as overexpression is more difficult in the presence of a large quantity of maternal mRNA. To test for the upstream function of GSK-3, we generated a membrane-tethered form of GSK3 (contains a Src myristoylation sequence at the N-terminus [25–27]) and expressed it in embryos. As opposed to untethered GSK-3, myr-GSK-3 led to strong activation of signaling known as the naked phenotype similar to *zw3* loss of function mutants (Fig 1A–1G). Epidermal cells expressing myr-GSK-3 made fewer denticles and denticle precursors much like GSK-3 mutants (Fig 1E–1G), whereas cells expressing untethered GSK-3 did make denticles (Fig 1B and 1C). Additionally, the level of Arm protein increased with myr-GSK-3 expression as compared to wild-type embryos (Fig 2A) suggesting that the pathway was being activated normally downstream of myr-GSK-3.

**Table 1:** Summary of cuticle phenotypes from various pathway mutants expressing the four forms of GSK-3.

	UAS-MyrGSK-3	UAS-MyrGSK-3KK-MI	UAS-GSK-3	UAS-GSK-3KK-MI
Wild-type	Naked (~100% N>100)	Wild-type (~100% N>100)	Wild-type (~100% N>100)	Wild-type (~100% N>100)
*wg* ^*IG22*^	Naked (~25% N = 143)	*wg* (~25% N = 110)	*wg* (~25% N = 90)	N.D.
*zw3* ^*M11-1*^	Naked (~100% N>100)	Naked (~100% N>100)	Wild-type (~25% N = 189)	Naked (~100% N>100)
*dsh* ^*v26*^	*wg* (~50% N>100)	*wg* (~50% N>100)	*wg* (~50% N>100)	*wg* (~50% N>100)
*arr* ^*2*^	*wg* (~50% N>100)	*wg* (~50% N>100)	*wg* (~50% N>100)	N.D.
*arm* ^*F1a*^	*wg* (~50% N>100)	*wg* (~50% N>100)	*wg* (~50% N>100)	N.D.
*arm* ^*XM19*^	*wg* (~50% N>100)	*wg* (~50% N>100)	*wg* (~50% N>100)	N.D.
*arm* ^*043A01*^	*wg* (~25% N = 98)	N.D.	Crumbs (~50% N = 201)	N.D.

For *dsh*, *zw3*, *arr*, and *arm* mutations embryos were maternally and zygotically (M/Z) mutant, whereas *wg* was only zygotically mutated. All crosses use second chromosome armGal4 except for *arr* mutants where third chromosome daGal4 was used. The *wg* phenotype refers to a *wg*-like embryo or a loss of signaling phenotype. Naked refers to a loss of denticles phenotype similar to ectopic Wg activation or loss of GSK-3. N.D. means that the experiment wasn't done. In *zw3* mutant embryos, little paternal rescue is observed with half the embryos being completely naked and the other half mostly naked, so the rescue must involve a significant return to wild-type patterning only observed in UAS-GSK-3 expressing embryos. For *dsh*, *arr*, and *arm* mutations, paternal rescue is complete with 50% of embryos returning to a wild-type phenotype.

In order to establish the level of construct expression (UAS-Zw3-HA and UAS-myr-Zw3-HA) in the embryos, we performed western blots with protein extracts from embryos expressing the two constructs and probed for the presence of both endogenous GSK-3 and the exogenous expressed constructs with a pan-GSK-3 antibody (Fig 2B). The blot demonstrates that expression of exogenous GSK-3 relative to endogenous GSK-3 is comparable, showing that in embryos GSK-3 is not over-expressed but rather expressed at a similar level to the endogenous gene.

In a control experiment, we generated a myr-GSK-3 kinase dead form where two lysines from the ATP binding domain are mutated inactivating the kinase activity (KK83-84MI)[24]. This form did not activate signaling, nor did it rescue GSK-3 mutants (Fig 2C and 2D). Cells expressing the kinase dead form of myr-GSK-3 KK-MI did form denticles similarly to untethered GSK-3 and in contrast to myr-GSK-3, demonstrating that kinase activity at the membrane is required for pathway activation.

### Myr-GSK-3 functions downstream of Wnt to activate signaling

**Fig 3:**
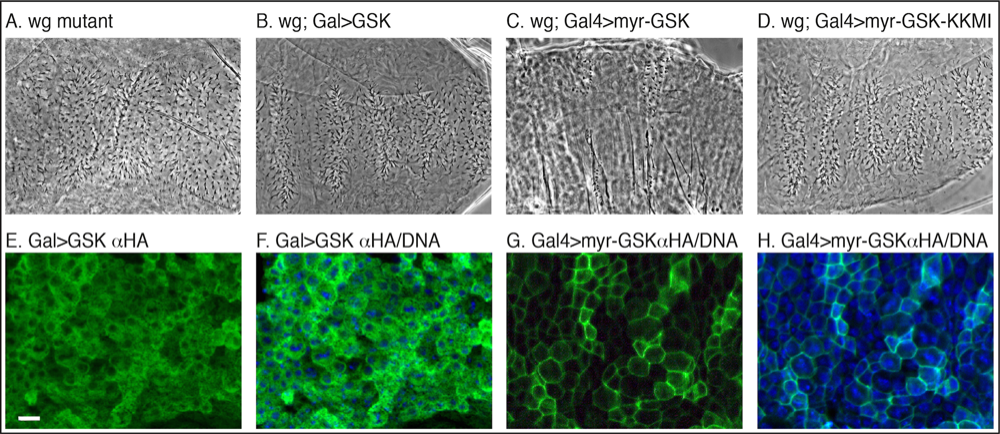
Membrane-tethered GSK-3 functions downstream of Wnt. (A) Cuticle of an embryo zygotically mutant for Wnt (*wg* ^*IG22*^) shows the classic segment polarity phenotype [29] and a loss of all naked cuticle. (B) Expression of wild-type GSK-3 using ArmGAL4 in Wnt mutants showed no effect, or the *wg* phenotype. (C) Expression of myr-GSK-3 in Wnt mutants led to the naked phenotype showing that myr-GSK-3 is epistatic to Wnt and functions downstream. (D) Kinase function is required as myr-GSK-3-KKMI failed to cause a naked phenotype in Wnt mutant embryos. Embryonic staining using ectopic tag HA in green and DNA in blue. (E-F) Close up of embryonic expression of GSK-3 shows high cytoplasmic expression. (G-H) Myr-GSK-3 localizes strongly to the plasma membrane. Scale bar = 10μm.

In Drosophila embryos the primary Wnt molecule responsible for patterning the embryo is Wingless (Wg or Wnt1) [28,29]. Wg binds to receptors on the plasma membrane beginning the formation of the activation complex, activating signaling and causing the naked cell fate [6]. All the events of signal transduction should therefore be downstream of Wnt, and we proceeded to test this by expressing myr-GSK-3 in *wg* mutants. We found that the absence of Wnt had no effect on the activity of myr-GSK-3 (Fig 3, Table 1), whereas untethered and kinase dead forms did not change the *wg* phenotype, showing that myr-GSK-3 is downstream of Wnt.

### Myr-GSK-3 requires Arrow to activate signaling

The membrane function of GSK-3 is to phosphorylate specific residues on the Wnt co-receptor Arrow (Arr, or Lrp 5/6 in vertebrates) [15,30–32]. When Wnt signaling is on, GSK-3 phosphorylates Arr providing binding sites for Axin and preventing the destruction complex from forming. As this function appears to be separate, we sought to place it genetically into the signal transduction pathway. We first looked at the localization of GSK-3 and myr-GSK-3 and found that the untethered form is predominantly cytoplasmic, but the tethered form is enriched at the plasma membrane of embryonic epithelial cells (Fig 3E–3H). The destruction complex role of GSK-3 is upstream of Arm, but downstream of Dsh and the receptors (Table 1, [18]). For example, a *dsh*, *zw3* double mutant gives a naked phenotype whereas a *dsh* single mutant shows a *wg* cuticle. This downstream function masks the upstream activating role genetically, but the myr-GSK-3 flies can now be used to overcome this limitation.

**Fig 4:**
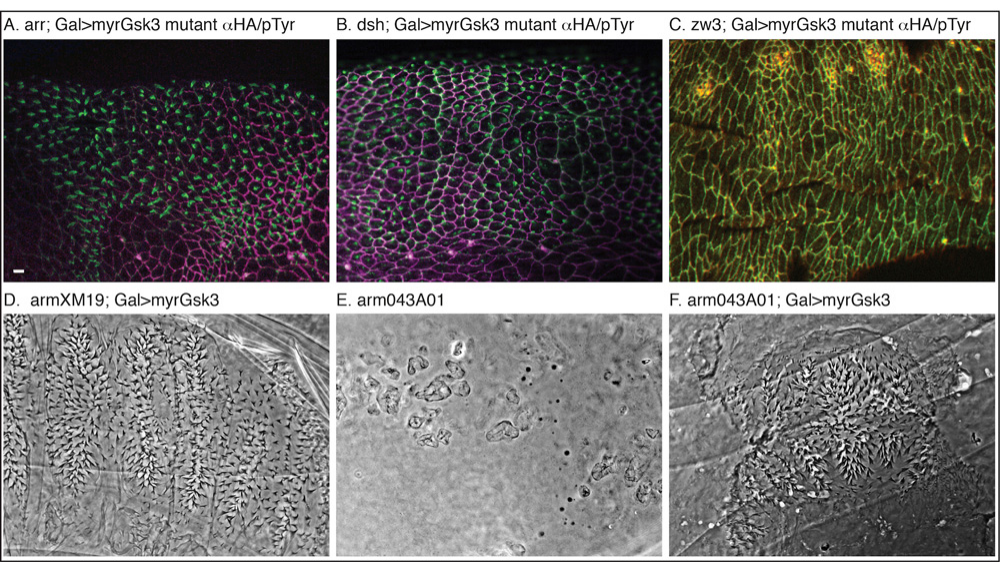
Myr-GSK-3 functions upstream of Arrow, Arm, and Disheveled. (A) *arr* ^*2*^ (M/Z) mutant embryos expressing myr-GSK-3 (HA magenta) fail to prevent formation of denticles (pTyr Green). (B) *dsh* ^*V26*^ (M/Z) mutant embryos expressing myr-GSK-3 (HA magenta) also fail to prevent formation of denticles (pTyr Green). (C) Expression of myr-GSK-3 in GSK-3 mutant embryos shows no effect as no denticles are formed (HA red, pTyr green). (D-F) Cuticles of an allelic series of *arm* mutants expressing myr-GSK3 show variable phenotypes. (D) Expression of myr-GSK-3 in *arm* ^*XM19*^ (M/Z) mutant embryos shows no effect. (E) *arm* ^*O43A01*^ (M/Z) embryos tend to fall apart leaving a *crumbs* phenotype. (F) Expression of myr-GSK-3 can rescue the cuticle integrity to a small degree. Scale bar = 10μm.

As the membrane complex function involves phosphorylation of Arr, we started by testing the interaction of Arr and myr-GSK-3. We made embryos maternally and zygotically mutant (dominant female sterile germline clones [33]) for *arr* and expressed myr-GSK-3 within them using the Gal4/UAS system [34]. Mutant *arr* (M/Z) embryos give a strong wingless-like phenotype [35], but are rescued by a paternal wild-type copy [35]. Expression of myr-GSK-3 had no effect in *arr* mutant embryos (Fig 4A), note denticle producing cells expressing myr-GSK-3. This experiment shows that Arr must be present for myr-GSK-3 mediated pathway activation.

### Myr-GSK-3 requires Dsh to activate signaling

Dsh is the upstream activating protein that inhibits the destruction complex. Genetically, it is upstream of GSK-3 and the destruction complex. Molecularly, its role appears to be in nucleating the activation complex at the membrane [36]. We investigated whether Dsh was required for myr-GSK-3 pathway activation. Loss of *dsh* in germline clone embryos (M/Z) mutants gives a strong wingless-like phenotype [10]. We expressed myr-GSK-3 in *dsh* (M/Z) mutant embryos but did not observe signal activation (Fig 4B), note denticle producing cells expressing myr-GSK-3. This experiment shows that Dsh is required for myr-GSK-3 mediated pathway activation.

As a final control, we also expressed myr-GSK-3 in *arm* (M/Z) mutant embryos in an allelic series of phenotype severity. In the two signaling loss of function alleles, *arm* ^*F1a*^ (milder form) and *arm* ^*XM19*^ (stronger form) [26,37] we did not observe any effect on patterning with expression of myr-GSK-3 (Table 1, Fig 4D). In the strong loss of function *arm* ^*043A01*^, however, where adherens junctions are disrupted and embryos fall apart during development (crumbs phenotype [38,39]), we did observe a mild rescue of cuticle integrity but no signaling activation (Fig 4E and 4F). These results show that Arm is required for myr-GSK-3 signaling activation, but additionally show that activation of the membrane-proximal complex can still inhibit degradation of Arm to the extent that adhesion function returns.

## Discussion

Recent findings in the Wnt signal transduction pathway have shown that the mechanism of this pathway is still not entirely understood. In this paper we focused on the genetics of GSK-3 and its two roles in the signal transduction pathway. We find that the activating role in the membrane signal-activating complex is conserved in Drosophila. We show that the activation occurs downstream of the extracellular ligand, but requires the membrane complex components Arr and Dsh to be present. This function is dependent on the kinase activity of GSK-3 as a kinase dead version cannot activate signaling. These results show the evolutionary conservation of this pathway from Drosophila to vertebrates.

**Fig 5:**
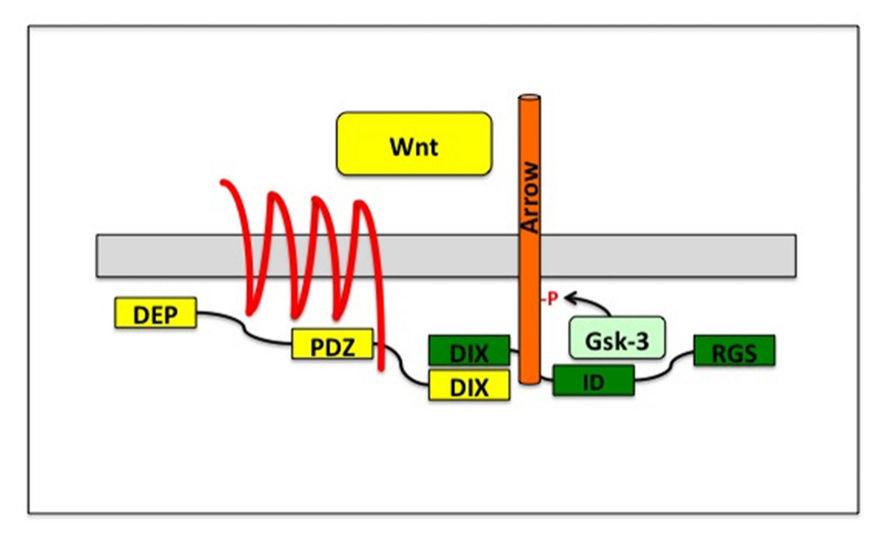
Simplified model for the membrane-proximal Wnt signaling activating complex. Wnt binding brings together the receptor Fz and the co-receptor Arrow/LRP5 or 6 extra-cellularly. Inside the cell, Axin and Dsh interact through their DIX domains bringing GSK-3 into proximity with phosphorylation sites on Arrow. In the absence of Dsh or Arrow, even membrane tethering of GSK-3 isn’t sufficient to activate Wnt signaling as the complex fails to form. ID on Axin is short for interaction domain, as this is the region mapped for CKI, GSK-3 and β-catenin interaction [57].

We are not able to answer the pressing question, however, as to how the destruction complex moves out of the cytoplasm, rearranges in the presence of Dsh/Arrow and activates signaling, a hot topic in the Wnt field [40,41]. Our results only show that both are required for the proper transduction of signals. From a genetic perspective, our findings formally show that GSK-3 kinase activity has two separable roles required for signal transduction. Myr-GSK-3 is targeted to the membrane through a lipid modification, where in the presence of Dsh and Arrow it activates signaling. If the role of the activating complex was simply to localize GSK-3 to the membrane, then Dsh and Arrow would be dispensable, but our epistasis experiments show this to not be true. We therefore believe that to be able to phosphorylate LRP5/6, GSK-3 requires the presence of complex components to facilitate phosphorylation (Fig 5). As our western blot shows, the expression levels achieved in embryos are not high compared to the endogenous GSK-3 expression, and certainly much lower than those achieved in tissue culture cells [15,40]. Similarly, we had previously failed to get strong Wnt activation in embryos with a membrane-tethered cytoplasmic domain of Arrow [17] whereas this worked very well in cultured cells [42]. Taken together, these results suggest that at the levels of expression achievable in Drosophila embryos, the membrane-proximal activation complex is required for membrane-tethered GSK-3 to be able to activate signaling.

Dsh is required under normal circumstances to activate signaling. It binds to Fz through the PDZ domain and to Axin through both their DIX domains [43,44]. Once the external binding of Wnt to both LRP and Fz is included, a five protein complex holds the receptors in place forming the activation complex (Fig 5). Interestingly, two studies argued that membrane tethering of APC and Axin is sufficient to inactivate signaling, or to reconstitute the destruction complex at the membrane [27,45]. It will be interesting to dissect the specifics of these models, as these results imply that simple membrane localization does not activate signaling, and suggests that it is the composition of the complex perhaps controlled through phosphorylation that determines whether signaling will be turned on or off [40]. Further, it is most intriguing that apart from GSK-3 other components including APC and Axin appear to have complicated roles in the pathway suggesting that much work remains before we fully understand the canonical Wnt signaling pathway [46–48].

## Materials and Methods

### Crosses and expression of UAS constructs

Maternally mutant eggs were generated by the dominant female sterile technique [49]. Oregon R was used as the wild-type strain. Please see Flybase for details on mutants used (flybase.bio.indiana.edu). Mutants used: *wg* ^*IG22*^, *zw3* ^*M11-1*^, *dsh* ^*V26*^, *arr* ^*2*^, *arm* ^*XM19*^, *arm* ^*F1a*^, and *arm* ^*O43A01*^ [37]. For mis-expression experiments, the ArmGAL4 2^nd^ chromosome and daGAL4 3^rd^ chromosome drivers were used. All X-chromosome mutants use FRT 101 except for *dsh* ^*V26*^ that has FRT 18E and second chromosome *arr* ^*2*^ mutants use the G13 FRT. The following crosses were conducted:

1. *zw3* ^*M11-1*^ FRT101/ovoD1 FRT101; arm-Gal4/+ females x UAS-myr-GSK-3
2. *zw3* ^*M11-1*^ FRT101/ovoD1 FRT101; arm-Gal4/+ females x UAS-GSK-3
3. *zw3* ^*M11-1*^ FRT101/ovoD1 FRT101; arm-Gal4/+ females x UAS-GSK-3 KK-MI
4. *zw3* ^*M11-1*^ FRT101/ovoD1 FRT101; arm-Gal4/+ females x UAS-myr-GSK-3 KK-MI
5. *arm* ^*F1a*^ FRT101/ovoD1 FRT101; arm-Gal4/+ females x UAS-myr-GSK-3
6. *arm* ^*XM19*^ FRT101/ovoD1 FRT101; arm-Gal4/+ females x UAS-myr-GSK-3
7. *arm* ^*O43A01*^ FRT101/ovoD1 FRT101; arm-Gal4/+ females x UAS-myr-GSK-3
8. *dsh* ^*V26*^ FRT18E/ovoD2 FRT18E; arm-Gal4/+ females x UAS-myr-GSK-3
9. *arr* ^*2*^ FRTG13/ovoD1 FRTG13; da-Gal4/+ females x *arr* ^*2*^/+; UAS-myr-GSK-3
10. *wg* ^*IG22*^, Arm-Gal4/+ x *wg* ^*IG22*^; UAS-myr-GSK-3

Most X chromosomes were marked with the *yellow* mutation or the balancers were marked GFP to simplify analysis. For all crosses more than 100 embryos were analyzed in multiple, separate experiments (n >100).

### UAS-transgenes and GAL4 driver lines

Two ubiquitous drivers were used for expression of transgenes: the weaker armadillo-GAL4 and the stronger daughterless-GAL4 [34]. UAS constructs were made using Gateway recombination (Invitrogen). Myristoylated constructs were made by adding a sequence identical to the NH2 terminus of *src* (MGNKCCSKRQGTMAGNI) to the NH2 terminus of GSK-3 by PCR. This sequence has proven to be very effective for membrane targeting of Arm [25–27,37]. The PCR products were then transferred by Gateway cloning (Invitrogen) into pUASg.attB with COOH-terminal 3XHA tag (A kind gift from J. Bischof and K. Basler, Zurich) [50]. Transgenes were injected into attP2 (Strain #8622) P[CaryP]attP2 68A4 by BestGene Inc. (California) [51]. Kinase dead GSK-3 was made by mutating lysines KK83-84MI in the ATP binding domain [24]).

#### Antibodies and Immunofluorescence

Embryos were fixed with Heat-Methanol treatment [52] or with heptane/4% formaldehyde in phosphate buffer (0.1M NaPO4 pH 7.4) [26]. The antibodies used were: anti-Armadillo (mAb N2 7A1, Developmental Studies Hybridoma Bank developed under the auspices of the NICHD and maintained by The University of Iowa, Department of Biological Sciences, Iowa City, IA 52242), anti-HA (ratAb 3F10 and mouse 12CA5, Roche), rabbit anti-Armadillo [53], phospho-tyrosine pY99 (Santa Cruz Biotechnology), anti-β-tubulin (E7, DSHB), and anti-Sexlethal (mAb M-14, DSHB). Staining, detection and image processing as described in [54].

#### Western Blotting

Embryos were selected for fertilization and developmental stage, lysed in extract buffer (50mM Tris pH 7.5, 150 mM NaCl, 1% NP-40, 1mM EDTA, 10% Glycerol, Complete Mini Protease, Sigma) or RIPA lysis buffer (Santa Cruz Biotechnology Inc.), the extracts were separated on 7.5% SDS-PAGE, and blotted as described in Peifer et al.[55]. To compare expression levels of endogenous and exogenous GSK-3, the embryo extracts were made in a similar manner and separated on SDS-PAGE (4–20%) and blotted using Rabbit Anti-Zw3 primary antibody [56].
